# *In situ* morphometric characterization of *Aframomum melegueta* accessions in Ghana

**DOI:** 10.1093/aobpla/plt027

**Published:** 2013-05-23

**Authors:** J. Amponsah, N. Adamtey, W. Elegba, K. E. Danso

**Affiliations:** 1 Graduate School of Nuclear and Allied Sciences, University of Ghana, PO Box LG 80, Legon, Accra, Ghana; 2 Nuclear Agriculture Centre, Biotechnology and Nuclear Agriculture Research Institute, Ghana Atomic Energy Commission, PO Box 80, Legon, Accra, Ghana; 3 Biotechnology Centre, Biotechnology and Nuclear Agriculture Research Institute, Ghana Atomic Energy Commission, P O Box 80, Legon, Accra, Ghana

**Keywords:** Characterization, cluster analysis, descriptors, morphometric traits, phenetic cluster, rhizome, stolon.

## Abstract

In spite of the huge economic importance of *Aframomum melegueta* in the herbal and pharmaceutical industries, its production is limited by lack of planting materials (propagules). The plant also lacks scientific descriptors, which has often led to misidentification with adverse health implications. We therefore aimed at developing a descriptor list to facilitate the identification of *A. melegueta* using 34 morphometric traits comprising 18 quantitative and 16 qualitative characters. The morphological traits showed that *A. melegueta* has a characteristic stolon that produces tillers instead of rhizomes. The unweighted pair group method with arithmetic mean using both the nearest-neighbour and complete-linkage methods based on the 34 morphometric traits clustered the eight accessions into two main groups based on ecological location. The accessions from the Eastern and Ashanti regions were separated at similarity coefficients of 0.822 and 0.644, respectively, with a highly significant discriminant function. The Eastern accessions were further clustered into red or yellow fruits at similarity indexes of 0.936 and 0.865 using the nearest-neighbour and complete-linkage methods, respectively. The present study has shown that morphometric traits of *A. melegueta* are greatly influenced by its ecological habitat. It is envisaged that the descriptor list developed coupled with a morphometric description would enhance its identification and utilization.

## Abstract (precis)

Misidentification of *Aframomum melegueta*, an important medicinal plant has huge adverse health implications. Thus, the scientific description and characterization of the accessions in Ghana was imperative. The study found the plant to bear creepy stolons which bear terminal bud for production of tillers instead of rhizomes which had earlier been reported by other investigators. All the accessions had a distinctive non storage bulbous structure at the base of the pseudostem. The UPGMA dendrogram clustered the accession into Ashanti and Eastern accessions based on ecological location. The Eastern accessions were sub-clustered in two groups based on fruit colour.

## Introduction

*Aframomum melegueta*, commonly referred to as grains of paradise, alligator pepper or melegueta pepper, is a very important member of the family Zingiberaceae. It is endemic in the tropical rainforest of West Africa, where it occurs in the coastal forest zone from Senegal to Cameroon (Lock *et al*. 1977; Weiss 2002). Although in Ghana the plant is endemic in the Atewa range in the Eastern region, it is also cultivated in the Ashanti, Brong Ahafo, Central and Eastern regions (Lock *et al*. 1977; Lock 1980). Variations in morphometric traits of *A. melegueta* have been acquired over the years due to co-evolution with its ecological habitat. The fruit colour may be red or yellow with a smooth surface that wrinkles when dried while the number of seeds per fruit also varies. For example, while Weiss (2002) reported that a fruit of *A. melegueta* contains 60–100 seeds, Simon *et al.* (2007) counted 1200–2000 seeds per fruit. All these variations are indicative of genotypic differences in a phenotypically heterogeneous species. Thus, characterization will lead to the estimation of true genetic diversity within the species and will also provide a complete description of the morphometric traits of the plant.

Besides its rich aroma, the seeds of *A. melegueta* are nutritionally rich, containing high concentrations of calcium, potassium, iron as well as vitamins (thiamine, riboflavin, niacin and ascorbic acid) that confer medicinal properties to the plant (Okwu and Okpara 2005). Consequently, the plant is used to cure dysentery, fever, measles, leprosy, excessive lactation, post-partum haemorrhage and male erectile dysfunction (Allas *et al*. 1995; Olowokudejo *et al*. 2005).

However, in spite of its huge economic potential in the food, herbal medicine and pharmaceutical industries, the species is still conserved *in situ* in the wild and on farmers' fields. While *in situ* conservation allows the maintenance and co-evolution of viable species in natural environments, it also exposes the species germplasm to both biotic and abiotic stresses. With rapid depletion of the tropical rainforest coupled with emerging climate change, *in situ* conservation poses a serious threat to the continuous survival of this economically important plant in the wild.

Furthermore, the lack of a standard descriptor list for *A. melegueta* has resulted in poor ethno-nomenclature and misidentification of the plant. Consequently, *A. melegueta* is often confused with *Amomum granum* [*paradisi*], also a member of the family Zingiberaceae (Anonymous 2010*a*), with disastrous health consequences in traditional herbal medicine. Consequently, we developed a morphometric-based descriptor list using that of *Elletaria cardamomum* (International Plant Genetic Resource Institute (IPGRI) 1994) as a guide for *in situ* characterization of *A. melegueta* accessions grown in Ghana. We also identified phenotypic traits that differentiate the accessions using discriminant analysis.

## Methods

### Sampling sites of accessions

**Figure 1.**
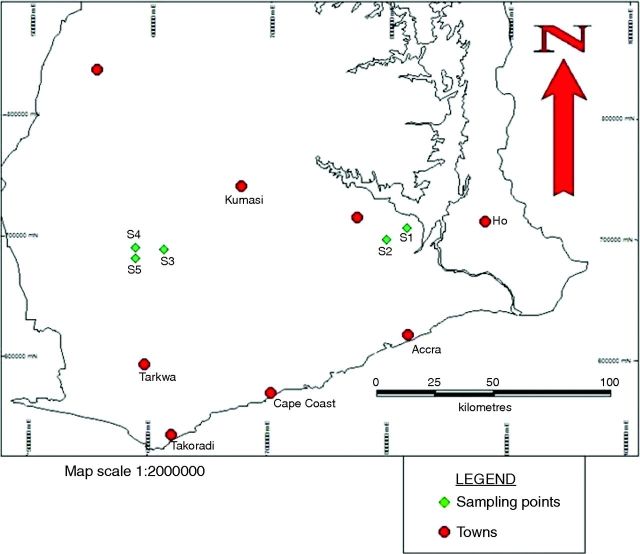
Geographical map of southern Ghana showing ecological areas where samples of *A. melegueta* were characterized *in situ*. A GPS was used to map out the sampling points and the points were fitted on a standardized map of southern Ghana.

The study was conducted *in situ* in five selected farming communities in the Eastern (Akanteng and Kobriso in West Akim District) and Ashanti regions (Sikaman, Brofoyedru and Maase Nkwanta in Obuasi Municipality) where *A. melegueta* is cultivated (Fig. 1). The communities lie within the tropical rainforest belt of Ghana with an annual rainfall ranging from 1250 to 1750 mm. The global positioning system (GPS) was used to locate the sampling points on the map. The soil types at Akanteng and Kobriso (Eastern region), and Sikaman, Brofoyedru and Maase Nkwanta were classified as Chromic Luvisol and Ferric Acrisol, respectively, according to the World Reference Base System of classification (Food and Agriculture Organization (FAO) 1998). These communities lie between latitudes 06°10″N and 06°24″N, longitudes 0°78″W and 01°72″W, and are elevated 161–312 m above sea level. Eight accessions comprising three from Akanteng, two from Kobriso and one each from Sikaman, Brofoyedru and Maase Nkwanta were used for the characterization studies. Five to 15 plants were chosen randomly from each accession; thus a total of 120 plants were used for the study.

### Development of the descriptor list for characterization

Thirty-four morphometric traits comprising vegetative, reproductive and yield characteristics were used to develop the descriptor list. Of these traits, 18 were quantitative while the remaining 16 were qualitative (Table 1). The descriptor list was based on that of *E. cardamomum*, which like *A. melegueta* belongs to the family Zingiberaceae (IPGRI 1994), as well as farmers' knowledge of the crop. Morphometric traits of *E. cardamomum* known to be non-discriminative and non-descriptive for *A. melegeuta* were not included in the descriptor list. Thus, only descriptive traits of *A. melegueta* were used as descriptors.

**Table 1.** Descriptor states of quantitative and qualitative traits used in the characterization of *A. melegueta*.

Qualitative traits			Quantitative traits
Descriptors	Descriptor state		Descriptors
	Designation	Interpretation	
Tiller colour	1	Crimson	Pseudostem per plant
	2	Dark red	
	3	Burlywood	Pseudostem height (cm)
	4	Green	
	5	Other (specify in the NOTES)	Pseudostem diameter (cm)
Stolon colour	1	Crimson	
	2	Dark red	Leaf number
	3	Burlywood	
	4	Other (specify in the NOTES)	Leaf length (cm)
Leaf shape	1	Linear	
	2	Lanceolate	Leaf width (cm)
	3	Oblong-lanceolate	
	4	Ovate	Phyllotaxy
	5	Other (specify in the NOTES)	
Pigmentation of midrib	0	Not pigmented	Panicle per plant
	1	Pigmented	
Presence of petiole	0	Absent	Panicle per tiller
	1	Present	
Presence of panicle	0	Absent	Nodes per panicle
	1	Present	
Inflorescence origin	1	Basal	Pedicel length
	2	Terminal	
Panicle habit	1	Prostrate	Sepal number
	2	Semi-erect (i.e. intermediate)	
	3	Erect	Petal number
Panicle branching	1	Non-branching	
	2	Branching	Stamen number
Flower type	1	Chasmogamous	
	2	Cleistogamous	Capsules per plant
Colour of calyx	1	Light green	
	2	Deep green	Capsule weight
	3	Other (specify in the NOTES)	
Colour of corolla	1	Yellow	Seeds per capsule
	2	Purple	
	3	Red	Seed weight
	4	Other (specify in the NOTES)	
Fusion of stamen	1	Free	
	2	Synandrous	
Capsule colour	1	Red	
	2	Yellow	
	3	State other	
Capsule shape	1	Globose	
	2	Ovoid	
	3	Narrowly ellipsoid to elongate	
Cross-section of capsule	1	Round	
	2	Angular	
	3	Ovate	

### Morphometric traits

Quantitative traits used for the characterization were the number of tillers per cluster of plants, the number of pedicels per cluster of tillers and the number of flower buds per pedicel. The number of sepals as well as seed weight and number of seeds per fruit were recorded. Additionally, the total number of leaves per plant, the average number of leaves in a longitudinal sequence before alternation (phyllotaxy), was recorded. The diameter of the tallest pseudostem as well as the height (from the base to the leaf axil of the fully opened distal leaf), leaf length and width at the broadest part of the fifth and sixth leaves were measured. The lengths of the pedicels were also measured and recorded as either <1 cm or >1 cm.

Qualitative data recorded were the basal colour of young tillers (3–8 months old) as well as fruit (capsule), stolon, corolla and calyx colours. These colours were described using the HyperText Markup Language colour chart (Anonymous 2010*b*). The presence or absence of panicle, petiole and midrib pigmentation as well as panicle habit and branching pattern were recorded. Furthermore, the shapes of leaf, capsule and capsule cross-section as well as flower type and fusion status were recorded. All qualitative data were standardized on a numerical scale using the IPGRI (1994) system of coding of qualitative data to allow for statistical analysis.

The data on colour traits and leaf shape were standardized on a scale of 1–5 (Table 1). The colours crimson, dark red, burlywood and any other, as well as the shapes linear, lanceolate, oblong-lanceolate, ovate and any other were coded as 1, 2, 3, 4 and 5, respectively (Appendix 1). One (1) or zero (0) was used to designate the presence or absence, respectively, of petiole, panicle and midrib pigmentation, while 1 and 2 designated basal and terminal inflorescence origin, respectively, as well as chasmogamous and cleistogamous flower types respectively. The various panicle habits (namely prostrate, semi-erect and erect) and panicle branching patterns were standardized using a scale of 1–3. The colour of the stolon, calyx, corolla and fruit (capsule) as well as fruit shape and cross- section, and leaf shape were also standardized accordingly.

All data were taken on 15 plants per accession for all traits except the yield, where only five plants per accession were used for the study. The data for each accession were averaged per number of tillers in the plants, and the averages were used to design a similarity data matrix for pairwise comparison between the accessions using unweighted pair group method with arithmetic mean (UPGMA) statistical methods.

### Cluster and discriminant analysis

UPGMA cluster analysis was performed using GenStat version 9.2 (Lawes Agriculture Trust 2007) to generate a dendrogram. Euclidean similarity coefficient or Euclidean distance was computed from the averaged morphological data for all the accessions. The nearest-neighbour linkage method based on the nearest-neighbour rule or the maximum similarity between two samples and the complete-linkage method based on the minimum similarity between two samples were used to cluster the accessions into groups. A two-group discriminant analysis was performed using SPSS version 16.0 (SPSS Inc. 2007) to determine the validity of the clusters as well as the discriminant traits. To determine how widely the accessions were separated from each other, group predictor variable means and standard errors were computed using the 34 morphometric traits.

## Results

### Morphometric traits and development of the descriptor list

**Figure 2.**
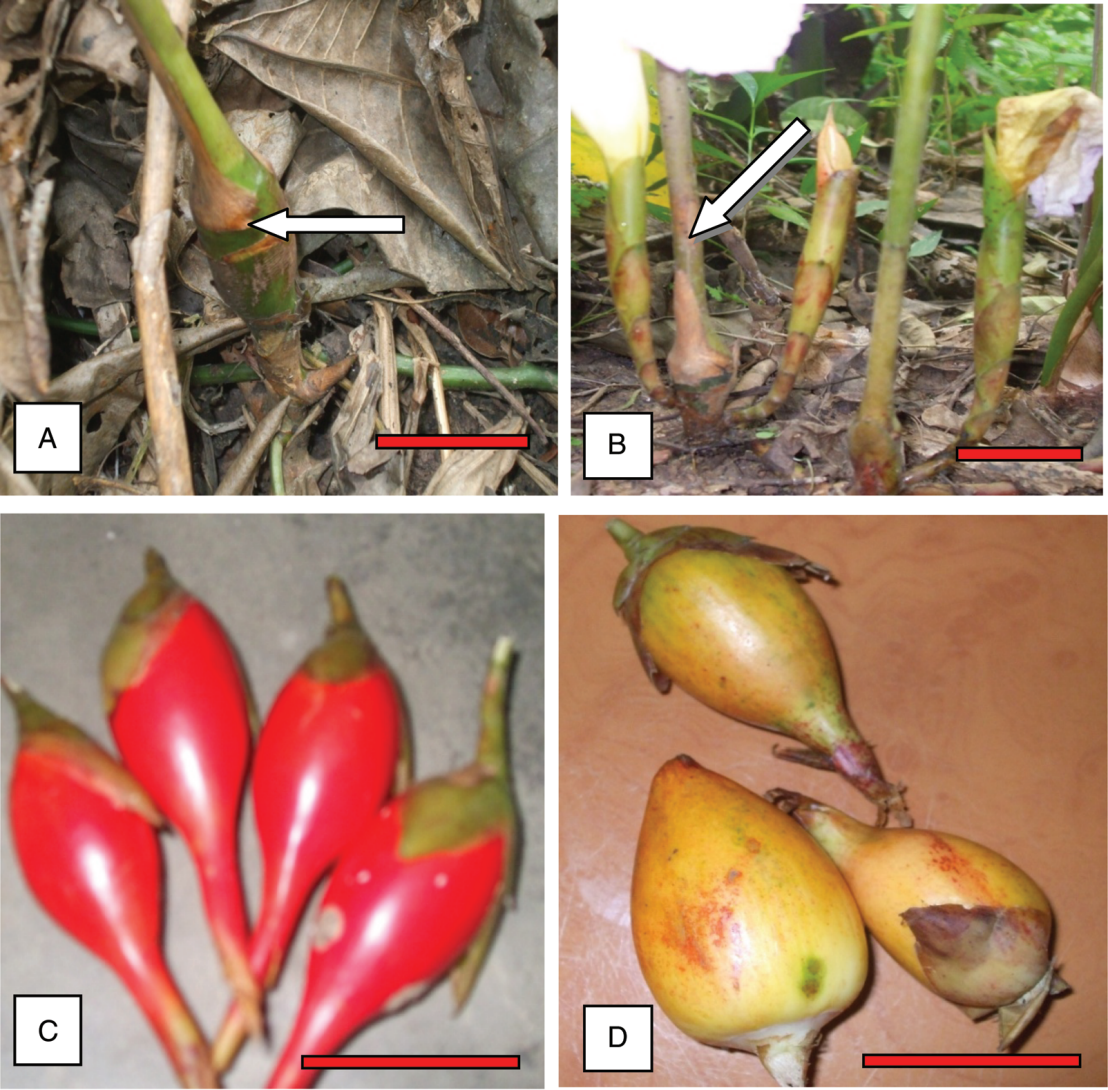
*Aframomum melegueta* plant showing (A) a bulbous pseudostem with brown leaf sheaths, (B) a pseudostem with two inflorescences at the base, (C) ellipsoid-shaped red fruits from the Ashanti region and (D) globose-shaped yellow fruits from the Eastern region (scale bars: A = 2 cm, B = 4 cm, C and D = 15 mm).

The varying morphometric traits used for *in situ* characterization of *A. melegueta* can be classified as either quantitative or qualitative (Tables 1 and 2). The variations depended on the location of the accession. The height of the pseudostem ranged from 157 to 183 cm with accessions from the Eastern region being significantly (*P* ≤ 0.05) taller (178 cm) than accessions from the Ashanti region (160 cm), indicating vigorous growth (Table 2). The pseudostem bulges just above the soil surface into a bulbous structure with either an ellipsoidal or globose shape, with differences in diameter depending on the locality (Fig. 2A). The bulbous structure from the Eastern region had a significantly (*P* ≤ 0.05) wider diameter (1.49 cm) than those from the Ashanti region (1.43 cm).

**Table 2.** Variations in morphological traits of *A. melegueta* accessions from the Ashanti and Eastern regions and Subgroups A and B. Pseu., Pseudostem; Caps, capsules or fruits; s, significant differences at *P* ≤ 0.05; ns, non-significant differences at *P* ≤ 0.05.

Characters	Eastern region mean	Ashanti region mean	Mean difference	Subgroup A mean	Subgroup B mean	Mean difference
Pseu. height	178.62 ± 9.43	160.40 ± 28.44	18.22^s^	176.2 ± 9.79	182.91 ± 7.39	6.7^ns^
Pseu. per plant	9.4 ± 2.95	7.53 ± 1.55	1.87^s^	8.25 ± 1.81	11.44 ± 2.13	3.19^ns^
Pseu. base colour	1.60 ± 0.82	4.00 ± 0.00	2.40^s^	1.06 ± 0.25	2.56 ± 0.53	1.50^ns^
Pseu. diameter (cm)	1.49 ± 0.11	1.43 ± 0.13	0.60^s^	1.51 ± 0.12	1.46 ± 0.08	0.05^ns^
Stolon colour	1.60 ± 0.82	1.00 ± 0.00	0.60^s^	1.06 ± 0.25	2.56 ± 0.53	1.50^s^
Number of leaves	28.6 ± 2.04	23.27 ± 4.62	5.30^s^	28.06 ± 2.05	29.56 ± 1.74	1.50^ns^
Leaf length (cm)	27.79 ± 1.26	25.23 ± 2.18	2.56^s^	27.61 ± 1.16	28.12 ± 1.42	0.51^ns^
Leaf width (cm)	4.87 ± 0.08	3.22 ± 0.35	1.65^s^	5.59 ± 0.58	3.55 ± 0.30	2.04^s^
Pedicels/pseu.	1.96 ± 0.61	1.80 ± 0.56	0.16^ns^	1.58 ± 0.62	2.11 ± 0.60	0.53^ns^
Pedicels per clump	7.72 ± 3.25	6.20 ± 2.18	1.52^ns^	6.31 ± 2.18	10.22 ± 3.42	3.91^s^
Caps per pseu.	1.64 ± 0.49	1.73 ± 0.46	0.09^ns^	1.56 ± 0.51	1.78 ± 0.44	0.22^ns^
Caps shape	2.00 ± 0.00	3.00 ± 0.00	1.00^s^	2.00 ± 0.00	2.00 ± 0.00	0.00^ns^
Caps colour	1.40 ± 0.50	1.00 ± 0.00	0.40^s^	1.00 ± 0.00	2.00 ± 0.00	1.00^s^
Caps diameter (cm)	5.50 ± 0.21	3.08 ± 0.13	2.42^s^	5.42 ± 0.19	5.66 ± 0.15	0.24^s^
Caps length (cm)	11.44 ± 0.23	10.09 ± 0.40	1.35^s^	11.37 ± 0.21	11.57 ± 0.21	0.20^ns^
Caps weight (g)	44.92 ± 1.31	24.85 ± 3.18	20.07^s^	45.11 ± 1.49	44.58 ± 0.88	0.53^ns^
Seeds per capsule	442.36 ± 12.34	348.00 ± 20.20	94.36^s^	436.44 ± 6.76	452.89 ± 13.54	16.54^s^
Seed weight (mg)	23.8 ± 0.60	17.54 ± 0.92	6.26^s^	23.88 ± 0.66	3.79 ± 0.47	0.09^ns^

Each pseudostem bears 2–4 brown leaf sheaths at the bulbous end, and numerous green leaves at the middle to the distal end. The number of leaves per pseudostem from Eastern accessions (28.60) was also significantly (*P* ≤ 0.05) higher than that for accessions from Ashanti (23.27). The older leaves close to the base of the pseudostem have a vestigial lamina that withers at maturity while those at the middle and distal ends have a distinct broad lamina and are either linear or lanceolate in shape with an alternate branching pattern.

**Figure 3.**
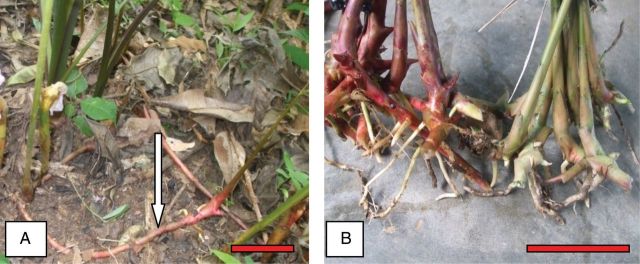
*Aframomum melegueta* plant showing (A) a creeping stolon (arrow) and (B) a basal pseudostem of crimson and burlywood in green tint colours (scale bars: A = 10 cm, B = 15 cm).

All accessions had fibrous roots and lateral buds at the base of the pseudostem. The lateral buds often develop into stolons that run on the surface of the soil, terminating in a bud. These terminal buds develop into a new shoot or cluster of shoots (Fig. 3A). Thus, *A. melegueta* has no rhizomes and tillering is initiated by stolon production. The accessions from the Eastern region produced 9.4 tillers per cluster, which is significantly (*P* ≤ 0.05) higher than accessions from the Ashanti region with 7.53 tillers per cluster. Consequently, the Eastern accessions form a comparatively denser cluster of tillers than the Ashanti accessions. The colour of the basal portion of the pseudostem and the stolon varied between accessions, ranging from crimson in accessions from the Ashanti region to dark red or burlywood in Eastern accessions (Fig. 3B). The basal colour of the young pseudostem is identical to that of the mother stolon; however, at maturity they all turn green irrespective of the ecological location.

The inflorescence arises from the base of the pseudostem (Fig. 2B) and develops into fruit as the plant matures. The accessions from the Ashanti region produced comparatively more fruit per shoot (1.73) than those from the Eastern region (1.64). The fruits varied in width, length, shape and colour (Fig. 2C and D), with fruits of accessions from the Eastern region having significantly (*P* ≤ 0.05) greater diameter and length than the Ashanti region accessions. Consequently, fruits of the Eastern region accessions had more seeds (442.36) per fruit than accessions from the Ashanti region (348.00).

The average seed weight of the Eastern region accessions (23.8 mg) was also significantly (*P* ≤ 0.05) higher than that of those from Ashanti (17.5 mg). The fruits of accessions from Ashanti were all red in colour and ellipsoid to elongate in shape, while those from the Eastern region had both red and yellow fruits that were either globose or ellipsoid in shape (Fig. 2C and D).

### Cluster analysis

**Figure 4.**
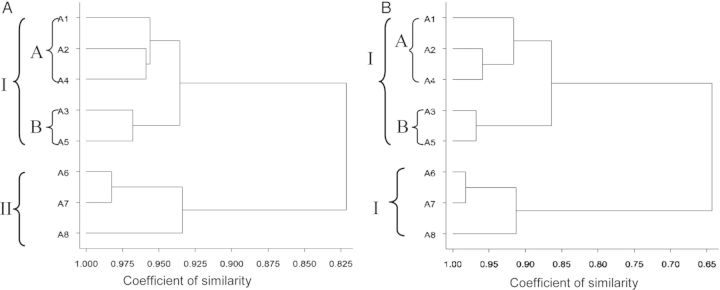
Nearest-neighbour (A) and complete-linkage (B) dendrograms of *A. melegueta* constructed from 34 morphometric traits.

The 34 quantitative and qualitative morphometric traits were used for cluster analysis and a dendrogram was constructed using the nearest-neighbour and complete-linkage methods (see Fig. 4). Both methods grouped the eight accessions from the two regions into two broad clusters based on ecological location, indicating the validity of the clusters. However, the similarity coefficients of the two clustering methods were different. The nearest-neighbour method separated Group I (Eastern accessions) from Group II (Ashanti) at a similarity coefficient of 0.822, while the complete-linkage method clustered the two groups at a similarity coefficient of 0.644. The Eastern accessions were further clustered into two subgroups (A and B) at similarity coefficients of 0.936 and 0.864 using the nearest-neighbour grouping and complete-linkage methods, respectively. Subgroup A comprised all accessions with red fruits from Akanteng and Kobriso (A1, A2 and A4), while Subgroup B comprised accessions with yellow fruits from Akanteng and Kobriso (A3 and A5), suggesting that the subgrouping was based on fruit colour and not ecological location.

The morphometric traits of the subgroups also varied. For example, the width of leaves (broadest part of the leaf) of Subgroup A accessions (5.59 cm) was significantly greater than that of Subgroup B accessions (3.55 cm). Contrarily, the fruits of Subgroup B accessions on average had a significantly (*P* ≤ 0.05) wider diameter (5.66 cm) than Subgroup A accessions (5.42 cm), and consequently produced significantly (*P* ≤ 0.05) more seeds per fruit (452.89) than Subgroup A accessions (436.44). However, the fruits of Subgroup A accessions were marginally heavier (45.11 g) than those of Subgroup B (44.58 g).

### Discriminant analysis

Of the 34 morphometric traits, only four, namely pseudostem basal colour, capsule colour, capsule diameter and capsule weight, were used by the standardized canonical discriminant function coefficient (DF1) to distinguish between Groups I and II. The Wilk's lambda of discriminant analysis performed to determine the significance and validity of the clusters as well as the morphometric traits used to predict group membership was highly significant (*P* ≤ 0.05), confirming that all eight accessions belong to two distinct subgroups of the same species. Similarly, the discriminant function (DF2) used to predict Subgroups A and B was also highly significant (*P* ≤ 0.05).

The standardized canonical discriminant function coefficient (Table 3) showed that the basal colour of the pseudostem had a predictive value of 1.754 and was the most significant trait for clustering the eight accessions into Groups I and II, followed by capsule colour (1.272), capsule diameter (0.826) and capsule weight (0.404). Contrarily, capsule colour was the only morphometric trait used for discrimination (Table 3) of the two subgroups.

**Table 3.** Standardized canonical discriminant function (DF) coefficient of *A. melegueta* traits with predictive values.

Predictor variables (traits)	DF1	DF2
Pseudostem base colour	−1.754	0.000
Capsule colour	1.272	1.000
Capsule diameter (cm)	0.826	0.000
Capsule weight (g)	0.404	0.000

## Discussion

Although morphometric traits are highly influenced by environmental factors, they have provided the basis for characterizing several plant species of economic importance. Thus, in this study we used morphometric traits to define variations in *A. melegueta* accessions. All the accessions had a swollen bulbous structure at the basal portion of the pseudostem whose size and shape differed significantly with ecological habitat. Accessions from the Eastern region had significantly (*P* ≤ 0.05) wider bulbous structures with a globose shape compared with those from the Ashanti region which had a smaller diameter and an ellipsoid shape. The swollen bulbous structure can be attributed to osmotic movement of water from the soil into cells as a result of the water potential gradient and therefore cannot be classified as a functional storage organ. Similar swellings with non-functional storage features have been reported in *Hornstedtia conica* (Maknoi 2009).

All the pseudostems studied had one or two creeping stolons. This observation is contrary to the report of Lock *et al.* (1977) and Weiss (2002) who independently claimed that *A. melegueta* has a rhizome. Arguably, rhizomes are underground modified stems that serve as sinks for storage of photosynthates and for vegetative propagation due to the presence of axillary buds. The stolons observed in *A. melegueta* had no lateral buds but a distal terminal bud that develops into a new shoot. Thus, the stolon bears neither a functional storage organ nor buds, and therefore cannot be classified as a rhizome as suggested by Lock *et al*. (1977) and Weiss (2002).

The basal colour of young tillers or pseudostems varied from crimson to burlywood, becoming green at maturity, and this may be attributed to the presence of anthocyanins. In banana and plantain, the pseudostem pigmentation varies from red to green and is an important morphometric trait for characterization (Irizarry *et al*. 2001; Queneherve *et al*. 2010). Thus, the pseudostem colour of *A. melegueta* could be a useful trait for characterization and identification of the plant by taxonomists.

There were also significant variations in fruit colour, length and number of fruits as well as the number of seeds per fruit. The number of fruits per plant in accessions from the Ashanti region (1.73) was comparatively higher than those from the Eastern region (1.64), although the Eastern region accessions had significantly larger fruit (5.5 cm) than the Ashanti region accessions (3.08 cm). Although we could not elucidate the exact reason for this observation, the low fruit set in the Eastern accessions could be attributed to noise from illegal mining activities that scared away potential insect pollinators (bumblebees). Sekercioglu *et al*. (2004) similarly reported that the low fruit set in *Rhynchantus beesianus* (of the family Zingiberaceae) in the Yunnan province of China was caused by the extinction of pollinators through habitat fragmentation. It is also possible that stiffer competition for assimilates among the fruits of the Ashanti accessions led to smaller fruit size and weight.

In determining the phenetic clusters or intraspecific relationships among the accessions, both the nearest-neighbour and complete-linkage methods separated all the accessions into two groups (Groups I and II) at similarity indexes of 0.822 and 0.644, respectively, based on ecological location of the crops. All Eastern accessions were clustered into Group I while the Ashanti accessions were in Group II. Subjecting the two phenetic clusters to discriminant analysis to authenticate the uniqueness of each group or cluster yielded a highly significant test statistic, a Wilk's lambda of 0.008 associated with the discriminant function (DF1), thereby confirming that Groups I and II are morphometrically distinct. Of the several morphometric traits, only pseudostem basal colour, capsule colour, capsule diameter and capsule weight had a highly significant standardized canonical discriminant function coefficient to separate the accessions into two major groups. The agro-ecological differences between the two regions may have had an influence on vegetative growth, seed production (yield), capsule and pseudostem base colour as well as fruit size.

Furthermore, the Eastern accessions were subdivided into two phenetic clusters at similarity coefficients of 0.936 and 0.865 using the nearest-neighbour and complete-linkage methods, respectively. Discriminant analysis of the clusters resulted in a discriminant function (DF2) with a highly significant Wilk's lambda (0.156), confirming that the two subgroups were unique. Although Subgroups A and B varied in a number of morphometric traits, the standardized canonical discriminant function coefficient revealed capsule (or fruit) colour as the only trait that subdivided the Eastern accessions into two clusters of distinct races.

## Conclusions

Our study has shown that there are two races of *A. melegueta* growing in the forest zones of Ghana that are distinct from each other in the colour of the fruits. Using the 34-morphological-descriptor list, a dendrogram separated accessions with red colour from those with yellow colour at similarity indexes of 0.936 and 0.865 using the nearest-neighbour and complete-linkage methods, respectively. We also observed that the plant has a stolon that bears a terminal bud and thus gives rise to tillers instead of rhizomes, as has been reported by other authors (Lock *et al*. 1977; Weiss 2002). The pseudostem of the shoot has a bulbous structure that has no functional storage properties. Although the morphological traits observed on the plant could be used for identification of the accessions, there is a need for molecular characterization of the plant to make its identification more authentic.

## Sources of Funding

The Government of Ghana provided the funding for this study.

## Contributions by the Authors

The main idea and topic of the study were formulated and proposed by K.E.D. and N.A. J.A. planned and conducted the research, which formed part of his M.Phil. thesis. W.E. also assisted in conducting the research and analysis of the data.

## Conflicts of Interest Statement

None declared.

## Supplementary Material

Additional Information

## Appendix 1 Descriptor list for characterization of *A. melegueta*

1.1 *PSEUDOSTEM HEIGHT*

Measured on the tallest tiller (up to the distal fully opened leaf axil). Average of five plants, 2–5 years old.

Average height in metres (cm) □

1.1.1 *Number of tillers (pseudostems) per plant*

Average of five plants, 2–5 years old □

1.1.2 *Tiller colour*

Observed at the base of the pseudostem

1 Crimson □

2 Dark red □

3 Burlywood □

4 Green □

5 Other (specify in the NOTES descriptor)

1.1.3 *Pseudostem diameter (cm)*

Average of five tallest pseudostems recorded 10 cm from the base □

1.1.4 *Stolon colour*

1 Crimson □

2 Dark red □

3 Burlywood □

4 Other (specify in the NOTES descriptor)

1.1.5 *Number of leaves*

Average of five plants, 2–5 years old □

1.1.6 *Leaf shape*

1 Linear □

2 Lanceolate □

3 Oblong-lanceolate □

4 Ovate □

5 Other (specify in the NOTES descriptor)

1.1.7 *Primary leaf length (cm)*

Average leaf lengths of the fifth and sixth leaves from a plant base of five plants, 2–5 years □

1.1.8 *Primary leaf width*

Average maximum width of the fifth and sixth leaves from the base of five plants, 2–5 years □

1.1.8.1 *Phyllotaxy*

Average number of leaves in longitudinal sequence before alternation

1.1.9 *Pigmentation of midrib*

Basal half

0 Not pigmented □

1 Pigmented □

1.1.10 *Presence of petiole*

0 Absent □

1 Present □

1.1.11 *Petiole length*

1 (<1 cm) □

2 (>1 cm) □

1.2 INFLORESCENCE AND FRUIT

1.2.1 *Presence of panicle*

0 Absent □

1 Present □

1.2.2 *Inflorescence origin*

1 Basal □

2 Terminal □

1.2.3 *Number of panicles per plant*

Average of five plants, 2–5 years □

1.2.4 *Number of panicles per tiller*

Average number of panicles per tiller of five 2- to 5-year-old plants □

1.2.5 *Number of flower buds per panicle*

Average of five plants □

1.2.6 *Panicle habit*

1 Prostrate □

2 Semi-erect (i.e. intermediate) □

3 Erect □

1.2.7 *Panicle branching*

1 Non-branching □

2 Branching □

1.2.7.1 *Panicle branching pattern*

1 Distal □

2 Entire □

3 Proximal □

1.2.8 *Flower type*

1 Chasmogamous □

2 Cleistogamous □

1.2.9 *Pedicel length*

1 <1 cm □

2 >1 cm □

1.2.10 *Colour of calyx*

1 Light green □

2 Deep green □

3 Other (specify in the NOTES descriptors)

1.2.10.1 *Number of sepals*

Average of five plants □

1.2.11 *Colour of corolla*

1 Yellow □

2 Purple □

3 Red □

4 Other (specify in the NOTES descriptors)

1.2.11.1 *Number of petals*

Average of flowers from five plants □

1.2.12 *Androecium: number of stamens*

1.2.12.1 *Fusion of stamen*

1 Free □

2 Synandrous □

1.2.12.2 *Fused stamen*

1 Filaments fused □

2 Anthers fused □

1.2.13 *Number of capsules per plant*

Average of five plants, 2–5 years old □

1.2.14 *Capsule colour*

1 Red □

2 Yellow □

3 State other

1.2.15 *Capsule shape*

1 Globose □

2 Ovoid □

3 Narrowly ellipsoid to elongate □

1.2.16 *Cross-section of capsule*

1 Round □

2 Angular □

3 Ovate □

1.2.17 *Number of seeds per capsule*

Average of 10 randomly selected capsules counted at third harvest □

1.2.18 Seed weight

Specify seed weight □
