# Supplementary material for: In situ morphometric characterization of Aframomum melegueta accessions in Ghana
Source: AoB Plants. 2013 May 23;5:plt027. doi: 10.1093/aobpla/plt027 (PMC3689184; doi:10.1093/aobpla/plt027)
Supplement: Additional Information [file supp_5_plt027_index.html]

In situ morphometric characterization of Aframomum melegueta accessions in Ghana — Additional Information 

# *In situ* morphometric characterization of *Aframomum melegueta* accessions in Ghana

## Additional Information

**Files in this Data Supplement:**

- Additional Information - Additional Information
