# Supplementary material for: In situ morphometric characterization of Aframomum melegueta accessions in Ghana
Source: AoB Plants. 2013 May 23;5:plt027. doi: 10.1093/aobpla/plt027 (PMC3689184; doi:10.1093/aobpla/plt027)
Supplement: Additional Information [file supp_plt027_plt027supp.docx]

Appendix 1: DESCRIPTOR LIST FOR CHARACTERIZATION OF *AFRAMOMUM MELEGUETA*

1.1.2 Pseudostem height

Measured on the tallest tiller (up to the distal fully opened leaf axil). Average of 5 plants, 2-5 years old.

Average height in meters (cm)

1.1.3 Number of tillers (pseudostems) per plant

Average of five plants, 2-5 years old

1.1.4 Tiller colour

Observed at the base of pseudostem

1Crimson

2 Dark red

3 Burlywood

4 Green

5 Other (specify in the NOTES descriptor)

1.1.5 Pseudostem diameter (cm)

Average of five tallest pseudo-stems recorded 10cm from the base

1.1.6 Stolon colour

1Crimson

2 Dark red

3 Burlywood

4 Other (specify in the NOTES descriptor)

1.1.7 Number of leaves

Average of five plants, 2-5 years old

1.1.8 Leaf shape

1 Linear

2 Lanceolate

3 Oblong-lanceolate

4 Ovate

5 Other (specify in the NOTES descriptor)

1.1.9 Primary leaf length (cm)

Average leaf lengths of the fifth and sixth leaves from a plant base of five plants, 2-5 years

1.1.10 Primary leaf width

Average maximum width of the fifth and sixth leaves from the base of five plants, 2-5years

1.1.10.1 Phyllotaxy

Average number of leaves in longitudinal sequence before alternation

1.1.11 Pigmentation of midrib

Basal half

0 Not pigmented

1 Pigmented

1.1.12 Presence of petiole

0 Absent

1 Present

1.1.13 Petiole length

1 (< 1cm)

2 (> 1cm)

1.2 INFLORESCENCE AND FRUIT

1.2.1 Presence of panicle

0 Absent

1 Present

1.2.2 Inflorescence origin

1 Basal

2 Terminal

1.2.3 Number of panicles per plant

Average of five plants, 2-5 years

1.2.4 Number of panicles per tiller

Average number of panicles per tiller of five 2-5 years old plants

1.2.5 Number of flower buds per panicle

Average of five plants

1.2.6 Panicle habit

1 Prostrate

2 Semi erect (i.e. intermediate)

3 Erect

1.2.7 Panicle branching

1 Non-branching

2 Branching

1.2.7.1 Panicle branching pattern

1Distal

2 Entire

3 Proximal

1.2.8 Flower type

1 Chasmogamous

2 Cleistogamous

1.2.9 Pedicel length

1 < 1cm

2 > 1cm

1.2.10 Colour of calyx

1 Light green

2 Deep green

3 Other (specify in the NOTES descriptors)

1.2.10.1 Number of sepals

Average of five plants

1.2.11 Colour of corolla

1 Yellow

2 Purple

3 Red

4 Other (specify in the NOTES descriptors)

1.2.11.1 Number of petals

Average of flowers from five plants

1.2.12 Androecium: number of stamen

1.2.12.1 Fusion of stamen

1 Free

2 Synandrous

1.2.12.2 Fused stamen

1 Filaments fused

2 Anthers fused

1.2.13 Number of capsules per plant

Average of five plants, 2-5 years old

1.2.14 Capsule colour

1 Red

2 Yellow

3 State other

1.2.15 Capsule shape

1 Globose

2 Ovoid

3 Narrowly ellipsoid to elongate

1.2.16 Cross section of capsule

1 Round

2 Angular

3 Ovate

1.2.17 Number of seeds per capsule

Average of 10 randomly selected capsules counted at third harvest

1.2.18 Seed weight

Specify seed weight
